# Commercial fishing gear modifications to reduce interactions between Atlantic sturgeon (*Acipenser oxyrinchus oxyrinchus*) and the southern flounder (*Paralichthys lethostigma*) fishery in North Carolina (USA)

**DOI:** 10.7717/peerj.2192

**Published:** 2016-07-20

**Authors:** Juan C. Levesque, Christian Hager, Eric Diaddorio, R. Jason Dickey

**Affiliations:** 1 Environmental Resources Management,Tampa,FL,USA; 2 Chesapeake Scientific, LLC,Williamsburg,VA,USA; 3 Greenville,NC,USA; 4 Natural Resources & Health Sciences Division, Cardno Inc.,Albany,NY,USA

**Keywords:** Bycatch, Commercial fisheries, Fishery interactions, Protected species

## Abstract

Bycatch of protected species in commercial fishing operations is a primary concern to fishery managers because it threatens the conservation, protection, and recovery of fragile species, such as the Atlantic sturgeon (*Acipenser oxyrinchus oxyrinchus*). One potential solution to reduce the risk associated with commercial fishing operations is to design commercial fishing gear that is more selective in terms of interactions between Atlantic sturgeon and commercial fisheries. Given this conservation and management need, the overarching goal was to reduce Atlantic sturgeon fishery interactions and maintain southern flounder (*Paralichthys lethostigma*) catch in North Carolina. The specific objectives of this study were to design and evaluate the effectiveness of a modified gillnet. Overall, the results proved that lowering the profile and amount of webbing had a beneficial impact at reducing Atlantic sturgeon incidental encounters and bycatch. The modified gillnet reduced bycatch and Atlantic sturgeon encounters by 39.6% and 60.9%, respectively. Our design entangled 51.6% fewer southern flounder, which corresponded to a 48.9% reduction in total weight; the modified gear entangled slightly larger southern flounder than the control gear. Our findings showed the number of Atlantic sturgeon encounters was positively associated with mean water depth, with more Atlantic sturgeon encountered in deeper (5.1–6.3 m) than shallower waters; 75% were encountered at depths between 4.6 and 6.1 m. Most southern flounder (*n* = 518, 39.7%) were taken at a water depth between 3.76 and 5.0 m. This observation suggests that southern flounder prefer slightly shallower waters than Atlantic sturgeon.

## Introduction

Bycatch in commercial fishing operations is one of the biggest challenges for fisheries managers tasked with conserving, protecting, and sustaining marine resources (Read & Rosenberg, 2002; Harrington, Ransom & Rosenberg, 2005; Read, Drinker & Northridge, 2005). The Magnuson–Stevens Fishery Conservation and Management Act (MSFCA, 1996) defines bycatch as “…fish which are harvested in a fishery, but which are not sold or kept for personal use, and includes economic discards and regulatory discards…”. Bycatch is a broad and complex term that has been used in the scientific literature in a variety of ways (e.g., Alverson et al., 1994), but in general, it can be defined as any aquatic organism (e.g., bird, fish, or mammal) that is incidentally captured (i.e., non-targeted; retained catch of non-targeted species) in some type of fishing gear (e.g., seines, trawls, and gillnets) or specialized equipment (e.g., hopper dredge) that is either retained or discarded back (portion of catch returned to the sea because of economic, legal, or personal reasons/choice) to the sea (Alverson et al., 1994; Graham, 2010); bycatch can be either dead or alive (Davis, 2002; He, 2015). It should be noted that all prohibited species (alive or dead) must also be discarded back to the sea (Alverson et al., 1994). Bycatch also includes various scenarios, such as captured and discarded, captured and retained, or regulatory discards (i.e., imposed regulatory measures (e.g., size, sex (male or female), time/space, or quota). Discards can even include marketable species. Given the availability of space on a vessel, sometimes commercial fishermen will use grading procedures; the discard of a marketable species to retain the same species or different marketable species at a larger size and price (Alverson et al., 1994). Alverson et al. (1994) indicated the term can also refer to unobserved mortality from contact/entanglement with fishing gear/equipment or catch associated with derelict fishing gear (i.e., ghost gear). Bycatch can include marine animals protected under the Endangered Species Act (ESA), Marine Mammal Protection Act (MMPA) or Migratory Bird Act. The survival rate of some discarded species (e.g., blacktip shark [*Carcharhinus limbatus*] and bonnethead [*Sphyrna tiburo*]) has been investigated (Hueter et al., 2006), but little is known about the survival of most protected species incidentally captured in fishing gears. Bycatch of protected species in commercial fishing operations is a primary concern to fishery managers because it threatens the conservation, protection, and recovery of fragile species. Due to strict regulations, it can also impact the economic sustainability of commercial fisheries because fishery managers are often forced to prohibit specific fishing gears and techniques (e.g., offshore drift monofilament gillnets). Many protected species have small populations and low reproductive rates (Hall, Alverson & Metuzals, 2000); thus, even small levels of mortality may prevent population recovery or lead to extirpation (Secor et al., 2002). One of the challenges for fishery managers is that most protected species display migratory behavior and undertake seasonal migrations that occur in conjunction with many economically valuable commercial fisheries, which compounds the problem (Lewison, Freeman & Crowder, 2004). Overlapping spatial and temporal distributions increases the risk and often leads to elevated fishery interaction rates. One potential solution to reduce interactions between protected species and commercial fishing operations is engineering fishing gear that is more selective.

Bycatch has been identified as a problem in the United States through various legislative actions (e.g., MSA (Magnuson-Stevens Act), ESA, MMPA), and substantial effort to reduce bycatch in commercial fisheries has been made over the last 20 years. However, most of the management and conservation measures have included time/area fishing closures, reductions in target quota, size-limits, fishing effort, and prohibition of specific fishing gear or fishing techniques (Harrington, Ransom & Rosenberg, 2005). Recently, some progress has been made in modifying commercial fishing gear (e.g., turtle excluder devices and circle hooks) and best management practices as a method to reduce bycatch of protected species, but additional research in this field is essential so fishery managers can improve how they manage protected resources while still achieving, on a continuing basis, the optimum yield for commercial fisheries (MSFCA, 1996). In many ways, this is a difficult and even unrealistic task for fishery managers given the stringent (i.e., jeopardy, potential biological removal, and zero mortality rate goal) requirements of the ESA and MMPA. Adding to the issue is that commercial fisheries continue to evolve, grow, and emerge (Levesque, 2010), so fishery management problems constantly change.

Under Section 118 of the MMPA, commercial fisheries are re-classified every year under the List of Fisheries process (Category I, II, and III; level of incidental mortality or serious injury of marine mammals), and additional species are classified as threatened or endangered under the ESA, such as the Atlantic sturgeon (*Acipenser oxyrinchus oxyrinchus*). In 2012, the National Marine Fisheries Service (NMFS) issued (6 February 2012) a final determination to list five distinct population segments (DPSs) of Atlantic sturgeon as endangered under the ESA (FR, 2012a; FR, 2012b): Gulf of Maine, New York Bight, Chesapeake Bay, Carolina, and South Atlantic. Despite this protection status, and a complete moratorium on possession of Atlantic sturgeon, these anadromous species are still incidentally captured in various commercial fisheries along the east coast of the United States, especially sink gillnet fisheries; sink gillnets are set along the bottom (Wirgin et al., 2015). Unfortunately, Atlantic sturgeon are particularly vulnerable to sink gillnets because they are a demersal species that feeds on benthic biota, such as polychaetes, crustaceans, and molluscs.

Given their anadromous life-history, Atlantic sturgeon are susceptible to numerous inshore commercial fishing operations (Logan-Chesney, 2013; Dunton et al., 2015) along the east coast of the United States, including commercial sink gillnet fisheries in North Carolina. In North Carolina, monofilament sink gillnets are used to target a variety of finfish (e.g., southern flounder [*Paralichthys lethostigma*]), which poses a threat to Atlantic sturgeon (Armstrong, 1999). Available scientific information indicates that commercial fisheries targeting southern flounder routinely encounter Atlantic sturgeon (White & Armstrong, 2000; FR, 2012a; FR, 2012b). Data on Atlantic sturgeon interactions with commercial fisheries in North Carolina is limited, but researchers have reported that Atlantic sturgeon mortality in gillnet fisheries in Albemarle and Pamlico Sounds is between 0 and 19%, and possibly higher (Armstrong, 1999; White & Armstrong, 2000). According to White & Armstrong (2000), a single commercial fisherman in the Albemarle Sound incidentally entangled 131 Atlantic sturgeon while targeting southern flounder with gillnet gear during 1998 through 2000. Atlantic sturgeon continue to be incidentally captured in North Carolina commercial fisheries, but updated fishery interaction information and a potential fishing gear/engineering solution are currently unavailable for the region. As such, the overarching goal of this study was to evaluate whether modifications to gillnet gear could reduce Atlantic surgeon interactions in the southern flounder fishery. The objectives were to evaluate the effectiveness of a modified gillnet in reducing Atlantic sturgeon fishery interactions and maintaining southern flounder catch. The specific objectives were to (1) describe, examine, and compare the bycatch associated with using a modified (experimental) versus a traditional (control) gillnet; (2) examine, compare, and test for differences in the number and mean size (length and weight) of southern flounder between a modified (experimental) and a traditional (control) gillnet; (3) examine, compare, and test for differences in the number and mean size (length and weight) of Atlantic sturgeon between a modified (experimental) and a traditional (control) gillnet; and (4) examine the environmental conditions (water depth and temperature) associated with Atlantic sturgeon encounters.

## Material and Methods

### Study area

**Figure 1:**
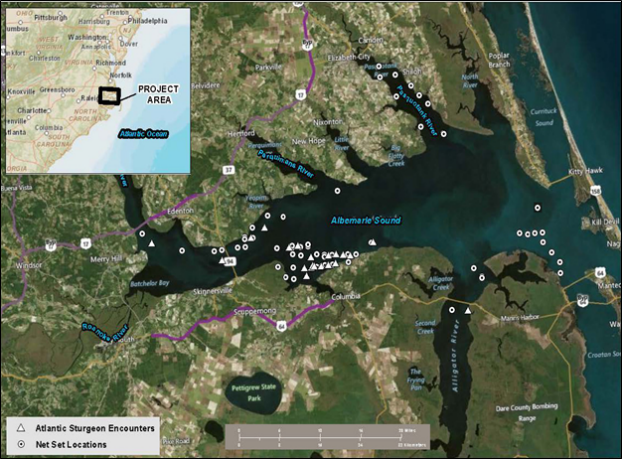
Map of the study area; Albemarle Sound, North Carolina (USA).

Based on historical fishing information, present commercial fishing effort for southern flounder, Atlantic sturgeon fishery interaction information, and recent discussions with state representatives and fishermen, we specifically conducted this study in Albemarle Sound, North Carolina (Fig. 1) near major rivers (Pasquotank, Perquimans, Chowan, Alligator, and Roanoke Rivers) to optimize the probability of encountering Atlantic sturgeon. We specifically selected this location because the largest Atlantic sturgeon commercial fishery once occurred in the Roanoke River, North Carolina (Kahnle et al., 1998), and Atlantic sturgeon continue to be incidentally encountered by commercial gillnet fishermen targeting southern flounder (Armstrong, 1999; White & Armstrong, 2000; FR, 2012a; FR, 2012b).

### Experimental and control gear specifications

To standardize the gear, the gillnet was constructed using traditional mesh size and lengths used by commercial fishermen targeting southern flounder in Albemarle Sound (Armstrong, 1999; J Levesque & Ms. Kathy Rawls, pers. comm., 2012, Ms. Kathy Rawls, North Carolina Division of Marine Fisheries (NCDMF); August 2012). It should be noted that the net and study were both designed prior to the release of revised commercial fishing regulations that prohibited the use of gillnets longer than 1,828.8 m (2,000) yards in Albemarle Sound; commercial fishermen can set more than one gillnet (large mesh (4–6.5 inch stretch mesh)) at a time, but the combination of gillnets cannot exceed 1,828.8 m. Another requirement is gillnets must be retrieved and re-deployed the following day when soaking overnight; commercial fishermen setting gillnets in Albemarle Sound, NC must “fish” or check their gilllnets once during a 24 hour period.

**Figure 2:**
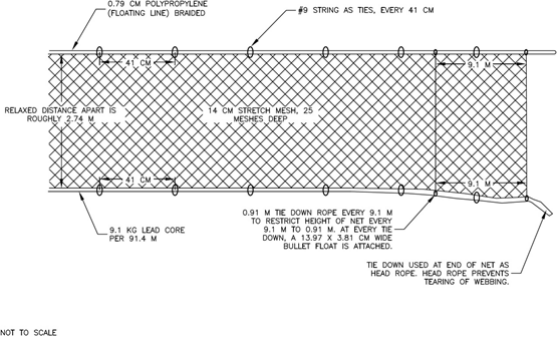
Control section gear specifications.

To ensure the monofilament gillnet was constructed using a typical technique for the region, a local experienced commercial fishermen was hired to design and assemble the gillnet. The monofilament gillnet was constructed with 30 equal length (91.4 m (100 yd)) panels or sections. The gillnet was constructed at a traditional length of 2,743 m (3,000 yd). The net design configuration used an alternating pattern approach (15 control and 15 experimental sections). Each control section was 91.4 m long, and it was constructed with 14.6 cm (5.75 in; 0.177 mm (diameter)) stretched mesh webbing hung on a 49.3% ratio. The panels were 25 meshes deep with a fishing height of approximately 3.1 m (10 ft) (Fig. 2). Every section had 0.91 m (3 ft) lines sewn in every 9.1 m (32.8 ft) that connected the leadline to the top or float line (tie-downs) at an interval of 6 meshes per tie. The float line was constructed with 0.79 cm (5/16 in) polypropylene braided line and 13.97 × 3.81 cm (5.5 × 1.5 in) floats were attached at the string ties every 9.1 m. The monofilament webbing was attached to the float line and leadline using #9 string ties every 41 or 43 cm (16–17 in). At the end of each section, a tie-down was sewn into the webbing as a head rope, which prevented the web from tearing. The bottom line was constructed using a 9.1 kg (20 lb) per 91.4 m leadline.

**Figure 3:**
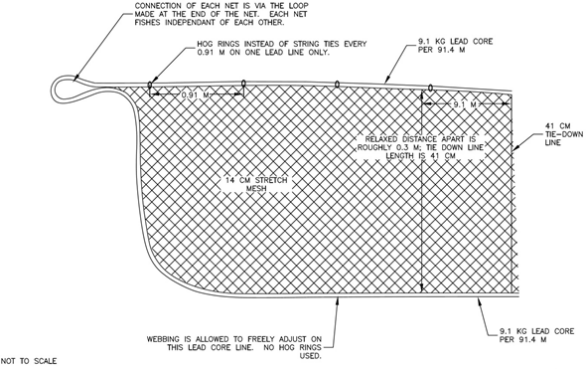
Experimental section gear specifications. It should be noted that the monofilament webbing in the experimental section did not hang in the water in a typical mode (i.e., vertical wall), it was more like a half moon or mushroom shape.

The experimental sections were each 91.4 m long and constructed of 14.6 cm (5.75 in; 0.177 mm (diameter)) stretch mesh hung on a 50% ratio. The panels were 15 meshes deep with a fishing height between 0.3 (1 ft) and 0.91 m (3 ft) (Fig. 3); the profile and amount of material (i.e., area) was approximately 75% less than the control sections. The tie-down lines were 41 cm long and sewn in every 9.1 m. Unlike the control sections, the top line of the experiment sections was replaced with another leadline (i.e., double leadline) to reduce the profile; no floats were used on the top line. Hog rings instead of string ties were used every 0.91 m (3 ft) on one of the top lead lines. The top and bottom line of the experimental section was constructed using a 9.1 kg per 91.4 m leadline. The monofilament webbing was hung through the lead core lines on the top and bottom rather than hung onto the net. One side of the webbing was pinned every 9.1 m and the other side was allowed to free float on the opposite lead core line. Unlike a typical vertical wall construction (i.e., control), the experimental section webbing formed a mushroom or half-moon shape, which reduced the height of the net. This design was based on the premise that reducing the height (75% reduction in comparison to the control) or profile of the net would reduce incidental encounters with Atlantic sturgeon, but still entangle sufficient numbers of southern flounder.

### Field procedures

Mimicking commercial fishermen that target southern flounder in Albemarle Sound, we mainly set the gillnet around sunset and retrieved it around sunrise (i.e., overnight sets). We also randomly conducted a few daytime sets because some fishermen sometimes set their gillnet during the day. It should be noted that fishermen in Albemarle Sound are legally allowed to soak their gillnet more than 24 h (overnight set); we mirrored the commercial activity as closely as possible by observing and discussing tactics with local fishermen. The net soak duration was calculated as the cumulative time between the beginning of the set and the end of the haul back; it was the entire time period the gillnet was in the water. The gillnet was generally deployed parallel to shore, but the direction was somewhat contingent upon the wind, current, and tide conditions. Every time the gillnet was deployed, the first panel was alternated between the control and experimental section to reduce any potential gear bias associated with distance from shore. The gillnet was secured to the bottom using 6.8 kg (15 lb) Danforth anchors attached to each end of the net for the duration of the set.

### Experimental study design

To optimize sample size and enable rigorous statistical evaluations of Atlantic sturgeon and southern flounder catch, field trials were conducted in April and during August through October (2014). Fishing effort and techniques closely mimicked the commercial fishery to reduce any potential sampling bias. In North Carolina, commercial landings of southern flounder usually peak in September and October (NMFS, 2014); therefore, we primarily focused our fishing effort during this period. However, because commercial fishermen sometimes target southern flounder during spring, a few sets were also conducted in April, 2014.

Sample size (i.e., number of sets) was estimated using historical Atlantic sturgeon fishery interaction rates and standard power analyses procedures. To detect various corresponding reductions (control vs experimental) in Atlantic sturgeon encounter rates (50–80%), we used the McNemar Test (*α* = 0.05 level); power curves were generated to estimate the number of sets necessary to achieve optimal power (i.e., sample size) according to gillnet length and historical Atlantic sturgeon encounters. Power curves were based on the mean annual Atlantic sturgeon catch rate (0.03 sturgeon/914 m of net/24 h soak) in Pamlico and Albemarle Sounds (NCDMF Observer Program (2001–2009) and White & Armstrong (2000)). Applying this power analyses approach, the number of sets necessary to detect an 80% reduction in Atlantic sturgeon encounters was 70.

Using a matched pair design (alternating experimental and control sections), the gillnet was randomly set within specific areas according to elevated historic Atlantic sturgeon fishery interactions and discussions with local commercial fishermen targeting southern flounder. The matched pair design helped ensure that both net sections had the same probability of encountering Atlantic sturgeon and southern flounder. Every time the gillnet was set, we alternated between deploying the control and experimental section. To reduce any potential bias associated with fishing experience, all sets were made by experienced fishery technicians with valid North Carolina commercial fishing licenses (i.e., commercial fishermen). To confirm quality control and scientific integrity, the Principle Investigator assisted with some of the field sampling.

Adaptive gear setting procedures were used for every set by considering Atlantic sturgeon and southern flounder daily catches. For instance, the gillnet was always set in ideal southern flounder and historical Atlantic sturgeon fishing grounds. Similar to standard commercial fishing techniques, the fishing location was altered if the catch was low. In general, fishing grounds were selected based on the environmental conditions (water temperature, depth, and current), discussions with local fishermen, and our fishing experience locating southern flounder in Albemarle Sound. Using adaptive procedures also ensured that an adequate sample size was obtained to allow for statistical inferences about the efficiency of the modified gillnet in terms of reducing Atlantic sturgeon encounters and retaining southern flounder; it also helped with reducing any potential sampling bias.

As previously stated, many fishermen set their gillnets in the evening and retrieve them in the morning (i.e., overnight set), but a few set and retrieve their gillnets at different times during the day depending on the southern flounder catch. As such, we used a similar approach with most of our fishing effort at night, and some during the day. Using this tactic, we elected to pool the data rather than to segregate (day vs night) it for analyses because it would have reduced our sample size and statistical power. We also chose this approach because we were interested in mimicking the fishing effort as closely as possible; segregating the data could have biased the results.

### Field data

The time, wind speed, wind direction, water depth, water temperature, and geographic coordinates (latitude/longitude) were recorded at the start and end of each set and haul. Catch (fish and crabs) was sorted, identified, and a representative sample was measured to the nearest millimeter in total length (TL). During the haul back, we measured and recorded the catch according to the corresponding net section (control and experimental). Southern flounder and Atlantic sturgeon were weighed to the nearest gram and measured to the nearest millimeter in fork length (FL) and TL. Atlantic sturgeon and other protected species (birds and sea turtles) were carefully handled and released as quickly as possible at the site of capture. Raw data were recorded on standardized data sheets and later reviewed for quality assurance by the field team lead, the Principle Investigator, and the data entry assistant. Data sheets were scanned, and then entered into Microsoft Excel^®^. Field sampling was carried out under the NMFS Bycatch Reduction Engineering Program (NA13NMF4720279) and the auspices of NMFS’s ESA Section 7 consultation process.

### Statistical analyses

Total count, percent occurrence, and percent total catch were calculated for each taxon. More detailed descriptive analysis was conducted for southern flounder and Atlantic sturgeon. Distributions of catch were plotted by section (control vs experimental) and individual species to assure that the most appropriate predictive models were used for analysis. All distributions were evaluated in terms of the best fit model: poisson, negative binomial, zero inflated negative binomial, or a zero-inflated poisson. If the criterion of normality was met, a one-factor analysis of variance (ANOVA) or a paired *t*-test was used to compare the catch, catch-per-unit-effort (CPUE), and size (length and weight) with respect to total catch, southern flounder, and Atlantic sturgeon. However, if the data did not satisfy the criteria for normality or it could not be transformed (logarithm, square root, or arcsine square root), then non-parametric procedures (Kruskal–Wallis, Wilcoxon signed-rank, and Mann–Whitney tests) were applied to evaluate the data. Catch-per-unit-effort was calculated as the number of individuals per one hour soak duration. Soak duration was defined as the elapsed time between the beginning of the set and the end of the haul. A Kolmogorov–Smirnov (KS) Goodness-to-Fit test was used to compare the distributions of Atlantic sturgeon and summer flounder (length and weight) by net section. The KS test was performed by computing the maximum distance between the cumulative distributions of the two samples. The Chi-square Goodness-of-Fit test was used to examine the representativeness of the sample for various categorical variables (e.g., water depth and water temperature) assumed to have uniform distribution. The Chi-square test was used to test the null hypothesis that the frequency of observed Atlantic sturgeon encounters was equal to the frequency of expected Atlantic sturgeon encounters. The Chi-square test was applied following the guidelines of Koehler & Larntz (1980); k classes > 3 (Zar, 1999). Firth regression was used to examine and evaluate probability of observing a positive catch of Atlantic sturgeon based upon a vector of covariates (net type, month, and water depth); firth regression is used to estimate parameter with small number of observations. All analyses were conducted using Microsoft Excel^®^ and Statgraphics Centurion XVI^®^ Version 16.1.

## Results

### Sampling effort and environmental conditions

Seventy sets (1,050 matched pairs) were conducted between April and October (2014) throughout Albemarle Sound; sets were often associated with major river mouths (Fig. 1). Nine sets (12.9%) were completed during 2–13 April, 2014, two sets (2.9%) on 31 August, 2014 and another 46 sets (65.7%) were completed during 3–20 October, 2014. In general, it took about an hour to set the net, but the retrieval (haul back) time varied; it was dependent upon the weather and other circumstances, such as net tangles and the time it took to remove the catch from the net. The net soak time duration ranged from 11.75 to 31.1 h with a mean of 23.1 h. In total, 192.02 km (210,000 yd) of net was set over a 75 day period, and the fishing effort (soak duration) was 1,615.87 h.

In April, the water temperature was between 10.6 and 15.4°C (51–59.8 °F) and the water depth was between 2.4 and 6.4 m (8–21 ft). The wind direction was generally north/ northeast, and the mean wind speed was 4.4 m/s (8.6 kn). In late-summer through fall (31 August–20 October), the water temperature ranged from 18.5 to 28.9°C (65.3–84 °F), and as expected, it decreased with time. The water depth varied between 0.7 and 7.0 m (2.4–23 ft) with a mean of 4.1 m (13.5 ft). Although we did not deliberately set the gillnet according to depth, the fishing effort was relatively evenly distributed (18–28%) by water depth. Thirteen sets (18.6%) were completed at a water depth between 1.3 and 2.5 m followed by 15 sets (21.4%) at a water depth between 2.6 and 3.75 m, and 19 sets (27.1%) at a water depth between 3.76 and 5 m. Twenty sets (28.6%) were completed at water depth between 5.1 and 6.3 m, and another 3 sets (4.3%) were completed at water depth between 6.4 and 7.5 m. The wind direction varied, but most of the days it was from the northeast direction (*n* = 31, 44%). The wind speed ranged between 0 and 12.9 m/s (0–25 kn) with a mean of 5.7 m/s (11.1 kn).

### Bycatch

The gear entangled 8,234 individuals representing 28 species in Albemarle Sound from April to October, 2014. The total catch consisted of 3,891 bony fish (23 species), 4,303 Atlantic blue crab (*Callinectes sapidus*), 35 rays (stingray [*Dasyatis* sp] and smooth butterfly ray [*Gymnura micrura*]), 3 double-crested cormorants (*Phalacrocorax auritus*), and 2 Kemp’s Ridley (*Lepidochelys kempii*) sea turtles. The control sections entangled 27 species, while the experimental sections entangled 20 species. The control sections entangled significantly more (24.7% [*n* = 1, 708]) individuals than the experimental sections (*t* (922) = 6.06; *p* < 0.01). Overall, the most numerically dominant fish entangled were Atlantic menhaden (*Brevoortia tyrannus*) (*n* = 2,046, 54%), southern flounder (*n* = 1,310, 35%), longnose gar (*Lepisosteus osseus*) (*n* = 129, 3%), and white catfish (*Ameiurus catus*) (*n* = 122, 3%).

The control (*n* = 4,316, 62%) sections entangled significantly more bycatch (excluding southern flounder) than the experimental (*n* = 2,608, 38%) sections (*t* (922) = 6.06; *p* < 0.05). Atlantic blue crab (*n* = 2,653, 62%), Atlantic menhaden (*n* = 1,307, 64%), and white catfish (*n* = 71, 1.6%) were entangled more often in the control sections, and Atlantic blue crab (*n* = 1,650, 54%), Atlantic menhaden (*n* = 739, 24%), and longnose gar (*n* = 51, 1.9%) were entangled more often in the experimental sections. The control sections entangled significantly more (9.9%) bony fish per set than the experimental sections (*t* (922) = 6.06; *p* < 0.05).

### Target species

In total, 1,310 (845.5 kg) southern flounder were taken during April through October, 2014. Most (*n* = 1,199, 92%) were taken from August to October, 2014. Overall, the experimental (*n* = 427, 33%) sections entangled 51.6% (*n* = 456) fewer southern flounder than the control (*n* = 883, 67%) sections (*t* (924) = 11.25; *p* < 0.01). The corresponding total weight was also significantly (*t* (924) = 12.35; *p* < 0.01) lower in the experimental sections (285.9 kg, 32%) than the control sections (559.6 kg, 66%). The CPUE for the control (0–44.9 southern flounder per hour, $\bar {x}=0$.64 southern flounder per hour) sections was greater than the experimental (0–21.8 southern flounder per hour with a mean of 0.31 southern flounder per hour) sections (*t* (923) = 11.18; *p* < 0.01).

The weight of individual southern flounder taken in the control section ranged from 0.2 to 8.3 kg with a mean of 1.08 kg. The corresponding total length ranged from 231 to 500 mm with a mean of 371.9 mm (*n* = 39). The weight of individual southern flounder taken in the experimental section ranged from 0.2 to 4.0 kg with a mean of 0.95 kg. The corresponding total length ranged from 332 to 492 with a mean of 393.9 mm (*n* = 29). Each net section entangled southern flounder with similar individual mean weight (*t* (819) = 0.72; *p* = 0.47), but the control sections entangled longer individuals (*t* (69) = − 2.13; *p* = 0.03). Evaluating the length and weight distributions, showed the experimental sections entangled southern flounder that were heavier (KS test, *D* = 3.09; *p* < 0.05) and longer (KS test, *D* = 1.88; *p* = 0.002) than the control sections.

Evaluating the total southern flounder catch by depth showed that most (*n* = 518, 39.7%) were taken at a water depth between 3.76 and 5 m. The remaining catch was distributed relatively evenly by depth category. Two hundred and forty-one (18.5%) southern flounder were taken at a water depth between 1.3 and 2.5 m, and 276 (21.2%) southern flounder were taken at a water depth between 2.6 and 3.75 m. Both sections (control and experimental) entangled around the same relative percentage of southern flounder by depth category; fishing effort was relatively similar among depth categories.

The price commercial fishermen received for southern flounder varied from $2.00 per 0.45 (1 lb) in late-September (2014) for medium size individuals to $3.25 per 0.45 kg (1 lb) in August (2014) for large size individuals. On average, commercial fishermen received around $2.56 per 0.45 kg for southern flounder during April through October (2014) in Albemarle Sound. Using the average price commercial fishermen received for southern flounder, the experimental sections entangled about $1,341 less than the control sections over the duration of the study.

### Protected species, Atlantic sturgeon

**Figure 4:**
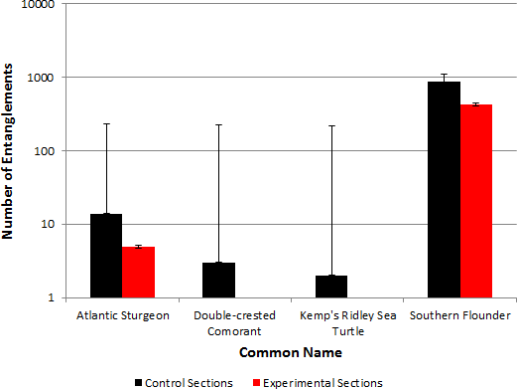
The total number of incidental encounters with protected species in association with the southern flounder catch by net section type in Albemarle Sound, North Carolina from April to October, 2014. The *y*-axis scale is a logarithmic scale (base 10). The error bars depict 5% error.

Thirty-seven individuals representing three protected species (Atlantic sturgeon (*n* = 32), double-crested cormorant (*n* = 3), and Kemp’s ridley sea turtle (*n* = 2)) were incidentally encountered during the study (Fig. 4); all protected species were released alive. No mortalities of Atlantic sturgeon were documented in either the control or experimental sections. It should be noted that two of the Atlantic sturgeon had external T-bar tags at the base of the left dorsal fin musculature (#49364 and #48022).

Seventy-two percent (*n* = 23) of Atlantic sturgeon were incidentally encountered in the control sections, but only 28% (*n* = 9) Atlantic sturgeon were incidentally encountered in the experimental sections (Fig. 4). Overall, the experimental sections incidentally encountered 60.9% (*n* = 14) fewer Atlantic sturgeon than the control sections (Wilcoxon signed-rank test, *Z* = 2.06; *p* = 0.04; *χ*^2^[1, 32] = 45.8; *p* < 0.001). Applying the McNemar test indicated 70 sets were necessary to detect a corresponding 80% reduction in Atlantic sturgeon encounters between the two net sections at an alpha of 0.05 and beta of 0.2 (80% power). Based on the number of sets completed, and the historical Atlantic sturgeon encounters with the fishery, results showed the difference between the two net sections was significant (*p* < 0.05; power > 80%). The CPUE for the control (0–0.1988 Atlantic sturgeon per hour, $\bar {x}=0$.0151 Atlantic sturgeon per hour) sections was greater than the experimental (0–0.75 Atlantic sturgeon per hour, $\bar {x}=0$.0059 Atlantic sturgeon per hour) sections (*t* (68) = 2.19; *p* = 0.03).

**Figure 5:**
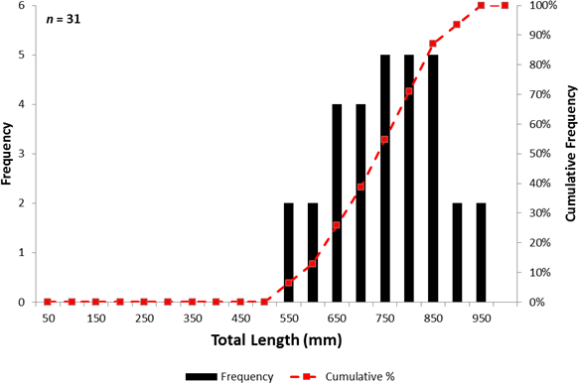
Length and cumulative frequency distribution of Atlantic sturgeon encountered in **Albemarle Sound, North Carolina from April to October, 2014.**

The overall length-frequency distribution of Atlantic sturgeon encountered ranged from 510 to 915 mm TL with a mean of 734 mm TL (Fig. 5). The total length of Atlantic sturgeon incidentally encountered in the control sections ranged from 510 to 915 mm with a mean of 738.6 mm. The corresponding weight ranged from 0.4 to 4.9 kg with a mean of 2.2 kg. The total length of Atlantic sturgeon incidentally encountered in the experimental sections ranged from 570 to 850 mm with a mean of 720.8 mm. The corresponding weight ranged from 1.0 to 2.9 kg with a mean of 1.8 kg. The control sections incidentally encountered Atlantic sturgeon with a similar length (Wilcoxon signed-rank test, *Z* = 82.0; *p* = 0.67) and weight (Wilcoxon signed-rank test, *Z* = 59.0; *p* = 0.38) as the experimental sections; the length (*χ*^2^ = 0.58; *p* > 0.05) and weight (*χ*^2^ = 0.39; *p* > 0.05) distributions were similar between the net sections.

In terms of entanglement location (Net Sections/Pair Number 1–30), 31% (*n* = 10) of the Atlantic sturgeon were incidentally entangled in either the beginning or end net sections. Despite this apparent difference, a Kruskal–Wallis test did not detect a significant difference in the ranks among the number of Atlantic sturgeon encountered by net section (*H* = 24.9; *p* = 0.68). The total number of Atlantic sturgeon incidental encounters ranged from 0 in August and October to 24 in September. In September (65.7% fishing effort), there were 16 and 8 encounters in the control and experimental sections, respectively. Despite the limited fishing effort in April (12.9%), there were 7 Atlantic sturgeon incidental encounters in the control sections and 1 encounter in the experimental sections).

**Figure 6:**
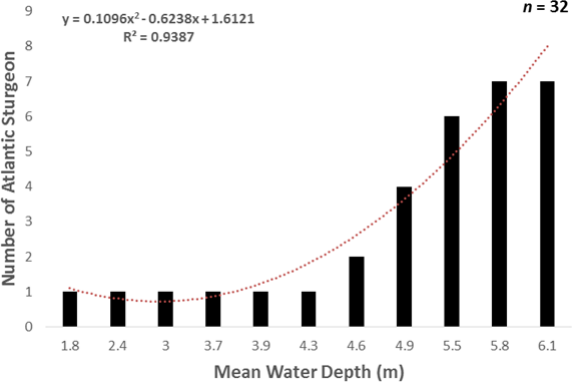
Number of Atlantic sturgeon incidentally encountered by mean water depth (m) in Albemarle Sound, North Carolina from April to October, 2014. The dashed line depicts the polynomial regression.

The fishing effort was distributed relatively similar by mean depth (1.3–6.4 m), but more Atlantic sturgeon were incidentally encountered in deeper than shallower waters (*χ*^2^[3, 32] = 25.2; *p* < 0.01). Twenty-four (62.5%) Atlantic sturgeon were incidentally encountered at a water depth between 5.1 and 6.3 m, which corresponded to 28.6% (*n* = 20) of the fishing effort. The number of Atlantic sturgeon incidentally encountered was positively associated with mean water depth, and it was explained by a quadratic polynomial regression (Fig. 6). A firth regression test did not find a significant interaction effect between the number of Atlantic sturgeon encounters and net section, month, or water depth. The best model fit did suggest that month and depth were significant predictors of a positive outcome for Atlantic sturgeon encounters.

## Discussion

Bycatch is a major issue for fishery managers around the world, particularly for those charged with preserving and recovering protected species. In the United States, one of the primary concerns is the incidental capture of protected species in commercial fishing operations, such as the long-lived Atlantic sturgeon. Although Atlantic sturgeon are protected under the ESA, and federal agencies are required to monitor incidental take, updated Atlantic sturgeon fishery interaction information is unavailable for most commercial fisheries (FWC, 2011). Unfortunately, only limited research has specifically focused on finding potential solutions to the Atlantic surgeon/fishery interaction problem in the United States. Most researchers to date have concentrated their research on understanding the life-history, movements, habitat preferences, and population dynamics of Atlantic sturgeon (e.g., Breece et al., 2013). Discovering a potential resolution to the fishery interaction problem is a major conservation and economic issue, especially since fishery managers are currently debating the continued authorization of sustainable commercial fishing activities, specifically those that interact with protected species (e.g., marine mammals, sea turtles, and Atlantic sturgeon). Demanding immediate attention, we conducted the first bycatch reduction study to evaluate modifications in commercial fishing gillnet gear to reduce interactions between Atlantic sturgeon and the southern flounder fishery in Albemarle Sound, North Carolina.

### Protected species; Atlantic sturgeon

Reducing incidental encounters (60.9%) of Atlantic sturgeon, our modified gillnet design seems to be a plausible solution to the Atlantic sturgeon/southern flounder fishery interaction problem in Albemarle Sound, North Carolina. Designing a monofilament gillnet with a reduced profile and associated webbing (<75% area) led to a statistically significant reduction in the number of incidental Atlantic sturgeon encounters. In addition, the experimental sections did not entangle any sea turtles or double-crested cormorants, unlike the control sections. Regrettably, the incidental encounters with sea turtles (*n* = 2) and double-crested cormorants (*n* = 3) were too low to make any conclusive statistical inferences.

Lowering the profile of the experimental net sections did not lead to any potential negative changes in the catch distribution of Atlantic sturgeon. The experimental sections incidentally encountered similar mean size, corresponding weight, and frequency distribution (length and weight) of Atlantic sturgeon as the control sections. Actually, these observations were consistent with what we anticipated given the design of our experimental net. We did not expect to detect any morphometric changes in the incidental Atlantic sturgeon encounters between the two sections since the experiment section was designed to reduce the numbers of encounters not necessarily the size or weight of the Atlantic sturgeon that were incidentally encountered; the control and experimental sections were constructed with the same mesh size. Overall, Atlantic sturgeon incidentally encountered in this study were slightly larger than those reported by Armstrong (1999). Using NCDMF (North Carolina Division of Marine Fisheries). Database (1990–1995), Armstrong (1999) reported that the number of Atlantic sturgeon encounters decreased with mesh size, but mean fork length increased with mesh size. It is unclear to us why the Atlantic sturgeon we incidentally encountered were larger than those previously reported, but it was probably related to the small sample size (*n* = 32) or limited number of sets. Despite the observed mean length for Atlantic sturgeon being inconsistent with previous studies, the control and experimental sections incidentally encountered Atlantic sturgeon with a similar length and weight distribution. As stated above, the net modifications we tested were not expected to influence the Atlantic sturgeon size/weight distribution, but rather the number of encounters. We are pleased that our modified gillnet did not influence the size/weight distribution because potential changes in length could have inadvertently harmed vulnerable size classes (<130 cm; minimum size-at-maturity (Van Eenennaam et al., 1996)). The incidental take of juvenile Atlantic surgeon has been reported to threaten the recovery of the population (Stein, Friedland & Sutherl, 2004). Though post-release mortality information is limited for Atlantic sturgeon, it is possible that smaller individuals incidentally taken in gillnet gear have a greater risk of mortality than larger individuals. Otter trawl gear is much different than gillnet gear in terms of characteristics, design, and application (passive vs active), but researchers in Nova Scotia, Canada did report the minimum post-release survival rate was high (94%) for Atlantic sturgeon (*n* = 29) incidentally encountered (Beardsall et al., 2013). Despite this high reported post-release survival rate, and relative similar size class (weir captures (112 ± 27 cm FL) and trawl captures [124 ± 15 cm FL) of captures, we simply cannot use these findings to infer the post-release survival of Atlantic sturgeon in sink gillnet operations given the major differences in the gear. However, we suspect that Atlantic sturgeon incidentally entangled in gillnet gear would have a higher mortality rate than those captured by bottom trawl gear given the extended soak times, but the comparison is difficult to make because mortality is dependent on many factors including, but not limited to, Atlantic sturgeon size, morphological location of entanglement (gills vs scutes), behavior, health, water temperature, fishing gear characteristics (soak time vs tow duration and tow speed) and other biological and environment conditions.

Most of the fishing effort (65.7%) and Atlantic sturgeon encounters occurred in September. Actually, after 30 September, 2014, we did not encounter any Atlantic sturgeon. It is difficult to presume why no Atlantic sturgeon were incidentally encountered after September since White & Armstrong (2000) incidentally encountered 69 Atlantic sturgeon from September to December (1998), and Armstrong (1999) incidentally encountered 20 Atlantic sturgeon in October (1998) and two in November (1998) in the same area. Perhaps it was related to changes in the environmental conditions and/or Atlantic sturgeon movement patterns. Armstrong (1999) hinted that Atlantic sturgeon may aggregate and increase swimming activity in Albemarle Sound during certain periods (spring and fall), so it is conceivable that many of the Atlantic sturgeon had simply moved to a different section in the sound (e.g., deeper waters) or even emigrated to coastal waters, depending on their size. Atlantic sturgeon emigration and movement (October) from river systems have been documented in other regions (Penobscot River, Maine) within their range (Fernandes et al., 2010), but in Albemarle Sound, juvenile Atlantic sturgeon are found throughout the year (Armstrong, 1999); seasonal movement information within the Albemarle Sound is limited. In general, seasonal movements are usually associated with tidal patterns and rising/falling water temperatures (Fernandes et al., 2010). It is also highly probable that Atlantic sturgeon have limited preferred habitat within Albemarle Sound (Armstrong, 1999), and preferred habitat could change seasonally, which decreases the likelihood of random encounters. Given this behavior and to reduce any potential sampling bias, we attempted to set the gear in ideal Atlantic sturgeon and southern flounder habitat by evaluating daily catch and discussing fishing success with local cooperative commercial fishermen. We used this approach because it is well documented that fishing success can potentially bias results in research studies focused on gear modification solutions. For instance, He & Jones (2013) reported significant differences in catch rates for Atlantic sturgeon between the two fishing vessels they used in their study. The researchers were able to consider this effect in their statistical analyses, but it should be noted that fishing tactics can bias results, especially if the experimental gear is deployed in a different way, area, or time than the control gear.

Despite the small sample size, the data showed that most of the Atlantic sturgeon incidental encounters were associated with deeper water. Many incidental encounters occurred at depths between 5.1 and 6.3 m. Relating the number of the Atlantic sturgeon encounters to southern flounder catch by depth showed that Atlantic sturgeon seemed to prefer slightly deeper waters in September; most southern flounder (39.7%) were taken in water depths between 3.75 and 5 m. We cannot be certain given the distribution of fishing effort and annual fluctuations in water temperature, but it is probable that Atlantic sturgeon prefer slightly cooler waters than southern flounder. If this is the case, then a potential best management practice to reduce interactions with Atlantic sturgeon could be for fishermen targeting southern flounder in Albemarle Sound to set their gillnet in more shallower water; we recommend testing this hypothesis in the future.

In many ways, the experimental sections out-performed previously tested gillnet designs in terms of reducing Atlantic sturgeon fishery interactions even though the nets used by other researchers were specifically designed for different fisheries (i.e., monkfish [*Lophius americanus*]). In earlier research, gillnet modifications designed to reduce Atlantic sturgeon fishery interactions resulted in conflicting and often mixed outcomes given the low statistical power, relatively high mortality rates for Atlantic sturgeon and other protected species, and high reduction in target catch (Fox et al., 2011). Building upon previous research, Fox et al. (2012) found that modifications to gillnet gear could provide a potential solution to Atlantic sturgeon encounters with large mesh sink gillnet fisheries in the mid-Atlantic and Northeast regions of the United States. Although their results were statistically non-significant, the research showed that incidental Atlantic sturgeon encounters could be reduced and landings of target species (monkfish and winter skate [*Leucoraja ocellata*]) could be maintained using a lower profile gillnet with tie downs. Fox et al. (2012) also found that incorporating specific tie-down configurations was important for maintaining target catch and reducing Atlantic sturgeon encounters in the monkfish fishery. In 2013, Fox et al., once again decreased the profile of the gillnet and compared it to the standard monkfish gillnet, but they were unable to achieve statistically significant reductions in Atlantic sturgeon encounters. The experimental gillnet did however catch similar numbers of monkfish and winter skate. Of note, Fox et al. (2013) found that most of the entangled Atlantic sturgeon were located in the upper half of the net, suggesting that a lower profile design might reduce more fishery interactions. He & Jones (2013) also discovered that a lower profile gillnet reduced Atlantic sturgeon incidental encounters in the monkfish fishery in Virginia and Maryland; although, the experimental nets caught significantly fewer monkfish (i.e., target species).

Given the findings from this present study and others, it appears that reducing the gillnet profile in the water, regardless of the fishery, has a beneficial significant impact on reducing the number of Atlantic sturgeon encounters. Unlike previous studies, our study was able to achieve sufficient statistical power to demonstrate a reduction in Atlantic sturgeon encounters. More importantly, the gear modifications in this study did not result in any observed mortalities of Atlantic sturgeon even though the modified gillnet had much greater mean soak duration than previous studies (Fox et al., 2011; Fox et al., 2013; He & Jones, 2013); soak duration has been correlated with Atlantic sturgeon mortalities (Fox et al., 2013). According to He & Jones (2013), every sturgeon encountered in the gear with soak duration greater than 24 h was dead. Fortunately, no mortalities occurred in Albemarle Sound during this study. Indeed, every Atlantic sturgeon incidentally encountered in the gillnet was in good condition despite warm water temperatures (>25°C, *n* = 16 encounters). We cannot be certain why they seemed to be in good health, but it is possible the Atlantic sturgeon got entangled in the gillnet just before haul back (i.e., just before sunrise).

Overall, our study was able to achieve more statistical conclusive results than previous studies due to the larger sample size (*n* = 70), the fact that the study was conducted independently rather than relying on various commercial fishing vessels, and the alternating section design (control and experimental sections). Previous researchers have often used more than one gillnet (e.g., He & Jones, 2013), which complicates the factors associated with fishing power and catchability. In our opinion, all of these factors helped us reduce potential sampling and gear bias. More importantly, the gillnet was specifically set in locations that were ideal for Atlantic sturgeon and southern flounder, underscoring the notion that fishing success can have on statistical inference and subsequent conclusions about the data.

### Southern flounder catch

The ultimate goal for researchers designing fishing gear is not only to reduce interactions between protected species and commercial fisheries, but to maintain the catch of specific target species (e.g., monkfish and southern flounder). Because southern flounder are an economically valuable commercial species in North Carolina, fishery managers need to ensure that any proposed gear modifications have little to no significant economic impact on the fishery before they consider implementing management measures. Our modified gillnet did reduce the number of incidental Atlantic sturgeon encounters, but the experimental sections did entangle significantly fewer southern flounder (numbers and corresponding weight) than the control sections. The experimental sections entangled 51.6% fewer individuals corresponding to a 48.9% loss in total weight of southern flounder than the control sections. Although statistically non-significant, the experimental sections entangled slightly larger individuals than the control sections, and the length and weight-frequency distributions were marginally different than the control sections. We would recommend future studies test a gillnet with slightly larger experimental sections (i.e., more meshes) to determine what size of gillnet reduces Atlantic sturgeon encounters, but still entangles sufficient numbers of southern flounder. He & Jones (2013) also reported modifications to gillnet gear yielded fewer monkfish smaller than 75 cm compared to the control net, but they did not detect a difference in monkfish larger than 75 cm. Fox et al. (2013) indicated they too entangled slightly smaller (statistically non-significant) monkfish in their modified gillnets.

Our research showed that reducing the profile of the gillnet had a negative economic impact on overall southern flounder landings. We acknowledge that commercial fishermen often operate at marginal profit levels, but considering the alternative options (e.g., permanently closing the fishery), the 48.9% loss in southern flounder catch (total weight) is relative in terms gross revenue, especially in comparison to other expenses, such as fluctuations fuel prices. The reduction in target catch in our study mimicked previous bycatch reduction studies that also modified the profile of the gillnet to reduce Atlantic sturgeon fishery encounters (Fox et al., 2011; He & Jones, 2013). Though our study cannot be compared to other studies given the fishery (southern flounder) and geographic location are completely different, the reduction in target catch was slightly greater than previously reported for other gear modification studies. Nevertheless, it appears that reducin g the profile of the gillnet tends to decrease the target catch regardless of the fishery. He & Jones (2013) found that changing the gillnet’s profile (number of meshes and tie-down length and spacing), decreased the landings of the main target species (monkfish) by 16.1%; but it had little effect on the secondary target species (winter skate). Fox et al. (2013) also reported fewer (4.5%) monkfish (target species) in their modified gear.

Modifying commercial fishing gear to reduce fishery interactions with protected species is challenging since many marine organisms have comparable preferred habitat, and aggregate or display similar movement patterns. Making modifications in fishing gear can either have no change in the target/protected species catch or it can alter catch of more than one species. Modifications in fishing gear or fishing practices can even have detrimental impacts to certain species. Fox et al. (2011) found that removing tie-downs did not have any impact on reducing Atlantic sturgeon encounters, but it did significantly reduced the target catch (monkfish) and caused a number of unacceptable marine mammal mortalities. Despite our findings demonstrating that reducing the profile significantly (statistically) decreased southern flounder landings, we believe this is still a potential viable solution for reducing Atlantic sturgeon encounters in the fishery given the escalading public, political, and environmental concerns associated with commercial fishery gillnets in North Carolina (Anonymous, 2016). Actually, based on several recent discussions with public representatives, there is growing momentum to prohibit the use of gillnets to target southern flounder in Albemarle Sound, North Carolina (Anonymous, 2016). Even if fishery managers were to consider our gear modification as a potential solution to the fishery interaction problem in Albemarle Sound, commercial fishermen would still need to evaluate whether they would be willing to or could afford to use the modified gear given the economic loss associated with the gear; fishermen only target specific species for economic reasons.

### Bycatch

Our modified gillnet was also successful at reducing bycatch by 39.6%. The total number of individuals entangled in the experimental sections was significantly lower than the control sections, which is encouraging given that bycatch is a major concern to commercial fishermen and fishery managers. In terms of biodiversity, the experimental sections entangled fewer species than the control sections. In general, both sections captured similar primary species, but there were some differences among a few species. The smooth butterfly ray, blueback herring (*Alosa aestivalis*), and channel catfish (*Ictalurus punctatus*) were only captured in the experimental sections, while the red horse sucker (*Moxostoma carinatum*) was only captured in the control sections. Currently, the blueback herring is a species of concern, so this could be an issue for fishermen and managers in the future even though it was one individual. Overall, species composition was similar between the two sections, which corresponded with previous studies (He & Jones, 2013). Given our gillnet design and the experimental approach (alternating sections), a difference in overall species composition was not expected. Modifying the gillnet had a positive effect on reducing bycatch and little to no effect on the entanglement of additional species, including protected species (endangered, threatened, or species of concern).

### Gear characteristics

Although our modified gillnet was successful at reducing Atlantic sturgeon fishery encounters, we found the modified gillnet did have some limitations in terms of its “fishability”. We define the term “fishability” as the way the fishing gear responds to the environmental conditions (i.e., winds, waves, and currents), which is very important to commercial fishermen because it affects catchability (i.e., number of fish captured and corresponding value). Commercial fishermen are always interested in catchability (i.e., optimizing catch while reducing fishing effort) because it affects profits, but one of their other main concerns is gear maintenance and associated costs. Often one of the drawbacks of using new gear or technology is its upkeep. To simulate the fishery, our modified gear was set under the same environmental conditions as local fishermen, which caused various issues (gear twists) that were related to incumbent weather (i.e., frontal storms). We noticed the experimental sections were often prone to twisting during high winds, currents, and waves. For example, on one occasion, after an overnight storm, the field crew had to untwist 732 m (800 yd) of net, which not only took longer to retrieve, but it probably increased the potential risk to Atlantic sturgeon since the gillnet was in the water longer. Although the field crew was able to untwist much of the webbing, gillnet repairs were occasionally required, which extended the haul back time. The other issue we noticed was that the modified gear was more fragile than the traditional gear in terms of its durability. By the end of the study, much of the monofilament webbing used in the construction of the experimental sections needed to be replaced given the occasional twists and tangles. Replacement of webbing translates to additional costs that would be a concern to commercial fishermen. In contrast, the monofilament webbing in the control sections was in much better shape.

Developing innovative fishing gear solutions requires refinement not only in terms of increasing target catch and decreasing bycatch, but reducing general gear maintenance and associated costs. In our opinion, the following refinements could increase the gear’s catchability (southern flounder landings) and fishability (lower gear upkeep time and costs): (1) adding an anchor every 457 m (500 yd) to minimize twisting; adding more anchors would secure the net to the bottom better; and (2) increasing the amount of drop back from 2 to 6 m in the experimental section webbing; increasing the amount of drop back should reduce the number of twists and increase southern flounder catch. We believe increasing the drop back in the experimental section webbing would reduce the tension between the top and bottom line causing the webbing to loosen, which would thereby entangle more southern flounder.

## Conclusion

Protected species interactions in commercial fisheries are a major problem in the United States, especially Atlantic sturgeon fishery interactions. One of the challenges for researchers interested in this topic is designing a suitable experiment that has a relatively low risk for the species of interest and a large enough samples size to conduct hypotheses tests and make statistical inferences with sufficient statistical power. Statistical power is influenced by significance criterion, the magnitude of the effect, and sample size. Given our power analyses approach that considered both the historical fishing effort and the number of Atlantic sturgeon encounters in the fishery, we were able to show a 60.9% reduction in Atlantic sturgeon encounters in the southern flounder fishery at a statistical power of 80%. As evident in this study, engineering solutions are possible for reducing Atlantic sturgeon fishery interactions, but modifications need to be fishery and location specific. Our study proved that reducing the profile and amount of monofilament webbing material (<75%) in the water can reduce the number of incidental encounters between Atlantic sturgeon and the southern flounder fishery in North Carolina, but further refinement is necessary in terms of gear specifics. Additional gear refining is necessary before commercial fishermen will support changing their traditional gear and tactics, especially if the transition to modified gear requires more maintenance. We mainly conducted the study in September to coincide with peak southern flounder fishing effort, but based on our limited fishing effort (*n* = 9) and associated catch (Atlantic sturgeon (*n* = 8) and southern flounder) in April, we recommend additional sets be conducted in the spring to further validate our results. The results also showed that more Atlantic sturgeon were incidentally encountered in deeper waters while more southern flounder were taken in slightly shallower waters. Additional research to investigate catch associated with depth is warranted. In summary, our finding are encouraging for Atlantic sturgeon conservation and maintaining sustainable commercial fisheries in Albemarle Sound, North Carolina, especially since fishery managers are now contemplating prohibiting gillnets in North Carolina waters.

##  Supplemental Information

10.7717/peerj.2192/supp-1 Supplemental Information 1 Raw Data

## Additional Information and Declarations

### Field Study Permissions

The following information was supplied relating to field study approvals (i.e., approving body and any reference numbers): We received permission from the North Carolina Division of Marine Fisheries, and the National Marine Fisheries Service (NMFS). A grant (NA13NMF4720279) was received from NMFS informing us to proceed with the study. We were given permission under NA13NMF4720279; this grant was our permit number.

### Data Availability

The following information was supplied regarding data availability: The raw data has been supplied as Data S1.
